# Construction of a Genome-Wide Copy Number Variation Map and Association Analysis of Black Spot in Jujube

**DOI:** 10.3390/plants14172782

**Published:** 2025-09-05

**Authors:** Yujia Luo, Zhi Luo, Cuiyu Wu, Lihu Wang, Fenfen Yan

**Affiliations:** 1 College of Horticulture and Forestry, Tarim University, Alar 843300, China; lluoyujia@163.com (Y.L.); lz15930225993@126.com (Z.L.); wcyby@163.com (C.W.); 2 The National-Local Joint Engineering Laboratory of High Efficiency and Superior-Quality Cultivation and Fruit Deep Processing Technology on Characteristic Fruit Trees, Southern Xinjiang Distinctive Foresty & Pomology Technology Innovation Center, Xinjiang Production and Construction Corps, Alar 843300, China; 3 College of Life Science and Technology, Tarim University, Alar 843300, China; 4 School of Landscape and Ecological Engineering, Hebei University of Engineering, Handan 056038, China

**Keywords:** Chinese jujube (*Ziziphus jujuba* Mill.), copy number variation (CNV), whole-genome resequencing, black spot disease

## Abstract

Copy number variation (CNV) is a common source of genomic structural variation by altering the number of DNA fragments, which in turn affects phenotypic variation and gene expression levels. However, there have been no reports of CNV in Chinese jujube (*Ziziphus jujuba* Mill.). In this study, we identified 16,570 CNVs from “Yuhong” × “Jiaocheng 5” and 140 hybrid progeny materials, of which 3607 CNVs were deletion type and 12,963 CNVs were duplication type. The distribution of CNVs in the Chinese jujube genome was systematically described, and the CNV genetic map of the whole genome level of the Chinese jujube hybrid offspring was constructed. Based on the field investigations, 13 individuals with severe black spot disease and no disease were analyzed for trait association. A total of 1837 CNVs were detected at the significant level of association, of which 1371 were duplication type and 466 were deletion type. And the GO (Gene Ontology) annotation item identified a systemic acquired resistance (SAR), and eight genes related to disease resistance were screened by the annotation. After validation by qPCR, these results further support the potential role in regulating black spot disease resistance. The constructed genome-wide CNV map of the hybrid progeny of Chinese jujube provides a new way of thinking for understanding the genetic basis of phenotypic variation of complex traits in Chinese jujube.

## 1. Introduction

Chinese jujube (*Ziziphus jujuba* Mill.) is a species of the genus *Ziziphus* in family Rhamnaceae, native to the middle and lower reaches of the Yellow River in China, with a cultivation history of over 7000 years [1]. Chinese jujube is not only rich in bioactive components such as polysaccharides, flavonoids, and cyclic adenosine monophosphate (cAMP)—which have been proven to regulate immune function, scavenge free radicals, and assist in blood glucose control—but also exhibits excellent adaptability and stress resistance in adversity [2,3]. Due to its excellent stress resistance, drought resistance, salt tolerance, high nutritional value and unique flavor, jujube has become the most widely cultivated dry fruit tree species in China. It is a unique fruit tree resource in China, accounting for over 98% of the world’s jujube resources [4,5]. In terms of geographical distribution across China, Xinjiang has the largest jujube production in China, particularly in southern Xinjiang, 88% of its cities and counties are jujube-producing areas, with a planting area of up to 473,000 hm^2^ [6]. Beyond Xinjiang, jujube cultivation is also widely distributed in other major regions: Hebei, Shandong, Shanxi, Shaanxi, and Henan [1]. With the rapid development of China’s jujube industry, issues such as the unreasonable structure of jujube cultivars, black spot disease, fruit shrink disease, and fruit cracking disease in main cultivars, as well as the lack of high-quality new cultivars in production, are becoming increasingly prominent. Therefore, to conduct research on jujube quality improvement and breeding and to cultivate new jujube cultivars with high yield, high quality, and excellent comprehensive resistance has become a key focus in genetic breeding [7].

With the development of high-throughput sequencing technology, next-generation sequencing (NGS) is widely used to directly resequence genomes [8,9]. Whole genome sequencing (WGS) is the most commonly used approach in NGS, involving sequencing different individuals within a species and identifying genetic variation between them when reference genome sequence information is available. These variations—such as single nucleotide polymorphisms (SNPs), insertion/deletions (InDels), structure variations (SVs), copy number variations (CNVs), among others—serve as molecular markers, thereby gradually enriching the scope of molecular breeding research [10]. Among these, CNV is a type of genomic structural variation, manifested as deletions and duplications of DNA fragments, and is a common form of genomic structural variation [11]. Abundant genetic variation in the genome is an important basis and source of phenotypic variation for various traits in organisms, and it influences phenotypic diversity and human diseases [12]. Over 20 years ago, studies revealed large-scale CNVs among human individuals, and researchers proposed that such widespread variations contributed significantly to genetic variation among populations [13,14]. This discovery has led scientists to extensively study CNVs. In 2006, Redon et al. [15] constructed the first-generation CNV map of the human genome, and identified 1447 CNV regions from 270 healthy individuals across 4 populations. These regions contained hundreds of genes, disease loci, functional elements, and fragment repeats. In recent years, the rapid development of genome sequencing technology has reduced the cost of CNV sequencing, and research on CNVs has also extended from humans to both animals and plants. Wang et al. [16] conducted CNV analysis using SNP genotyping data from 857 pigs, detecting a total of 312 CNVRs and functionally annotating the genes covered in these regions. Nandolo et al. [17] performed WGS sequencing on multiple African goat breeds and constructed a genetic map of CNVs in African goats. They identified a total of 253,553 CNVs, including 244,876 deletions and 8677 duplications. Additionally, 6231 CNVRs were identified, encompassing numerous genes that were significantly enriched in important biological functions, molecular functions, and cellular components. Compared with CNV research in humans and animals, studies on CNV in plants remain limited. Maize was the first plant species widely used for CNV research, and Springer et al. [18] identified structural variations in the maize genome using CGH technology, revealing extensive structural variations, including CNV and presence/absence variation (PAV). Subsequently, similar studies have been reported in *Arabidopsis thaliana* [19], barley [20], wheat [21], sorghum [22], rice [21,23], soybean [24,25], cucumber [26], sacred lotus (*Nelumbo nucifera*) [27], papaya [28], poplar [29], kiwifruit [30], Japanese plum (*Prunus sclicina Lindl*.) [31], and other plants, indicating that CNV contributes to research on genomic genetic diversity. With advances in molecular biology and sequencing technologies, numerous studies have demonstrated that CNV, as a major form of genomic genetic variation, plays a critical role in shaping phenotypic variation in complex human diseases and key economic traits in animals and plants [32,33]. Fernandez et al. [31] investigated three different plum varieties to explore CNVs in key softening-related genes—those that regulate ethylene perception and signal transduction during fruit ripening—aiming to identify CNVs associated with ripening differences. An increasing number of CNVs have been accurately detected, and the regulatory role of CNV in phenotypic traits and disease occurrence has emerged as a research hotspot.

Research showed that an anthracite black spot disease was discovered on the leaves of *Zizyphus mauritiana* Lamk. and *Zizyphus spinosus* Hu. [34]. Later, Zhao et al. [35] named this disease of Chinese jujube “fruit black spot disease”. Following a series of studies [36,37,38,39,40], it was established that the pathogen of Chinese jujube black spot disease was *Alternaria alternata*. After jujubes are infected with pathogenic bacteria, jujube black spot disease primarily affects jujube fruits and leaves. During the late white-ripening stage, the disease develops on the top or navel of the fruits. The flesh beneath the lesions is yellowish-brown and sponge-like, it eventually shrinks and spreads to the entire jujube [38], seriously impairing the commercial value of jujube fruits and causing significant losses to jujube growers.

Chinese jujube black spot disease is widespread and seriously hinders the healthy development of the jujube industry. In this study, using WGS technology, we sequenced the whole genome of an F_1_ hybrid population derived from the male-sterile cultivar “Yuhong” and “Jiaocheng 5” (characterized by high phytoplasma resistance and large fruit). The distribution of CNVs in the Chinese jujube genome was systematically characterized, and the CNV genetic map of the whole genome level of the Chinese jujube was constructed for the first time. Correlation analysis between the black spot trait and the functional annotation of genes encompassed by these CNVs was performed. Integrating relevant research, we identified candidate genes associated with the jujube black spot trait. The constructed genome-wide CNV map not only facilitates the molecular genetic improvement of economic traits but also enhances understanding of genetic variation in the jujube genome.

## 2. Results

### 2.1. Results of Resequencing Data

We utilized whole-genome resequencing data from the “Yuhong” × “Jiaocheng 5” cross and 140 F_1_ hybrid individuals, which were generated in our previous study [41]. The average sequencing depth of the parents was 12.43×, while that of the offspring was 2.62×. Sequencing was performed on an Illumina HiSeq^TM^ platform. A total of 203.02 G raw data were generated, with an average of 1.43 G per sample, and the Q30 base quality ratio reached 92.06%. We mapped these data to the jujube reference genome and found an average mapping efficiency of 97.09% and an average coverage depth of 2.76×, and that 73.33% of the reference genome was covered. These results indicate that the data are dependable, reliable, and of sufficient quality for CNV detection.

### 2.2. Genome-Wide Detection of CNVs

Genome-wide CNV detection was performed on the resequencing data of “Yuhong” × “Jiaocheng 5” cross and 140 F_1_ hybrids using CNVnator software (https://github.com/abyzovlab/CNVnator) [42], based on the read depth (RD) method. There were 2646 CNVs in the female parent “Yuhong”, including 1134 deletions and 1512 duplications; a total of 2533 CNV events were detected in the male parent “Jiaocheng 5”, with 1045 deletions and 1488 duplications (Table 1).

There were 16,570 CNVs in the entire F_1_ population, including 3607 deletions and 12,963 duplications, with an average of 118.36 CNVs, 25.76 deletions, and 92.59 duplications per individual. Regarding CNV length, the size range was 1-42,390,600 bp. We found that the number of duplication events was much higher than that of deletion events, with the number of deletions being 2.6× lower than that of duplications (Table 1).

### 2.3. Number of CNV Chromosomes

CNVs detected in hybrid offspring were screened. Those with *p*-values (denoting the significant difference between the sequencing depth of CNV regions and average genome-wide sequencing depth, denoted as *p*) greater than 1 were excluded, and high-reliability CNVs were retained for further analysis.

**Figure 1:**
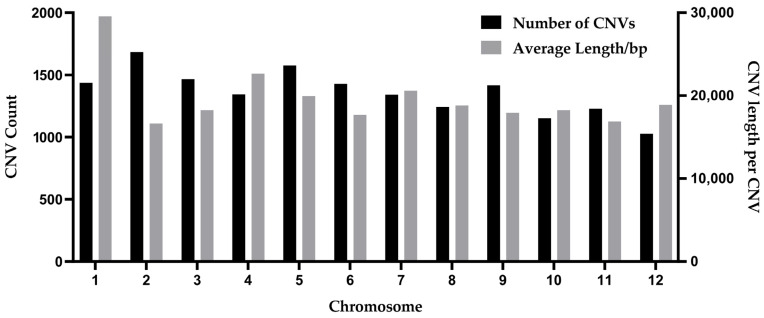
Characteristics of CNV distribution on chromosomes in jujube. Left: distribution of CNV count. Right: distributions of CNV length per CNV.

Finally, a total of 16,334 CNVs were identified, including 3378 deletions and 12,956 duplications, with an average of 24.13 deletions and 92.54 duplications per individual (Figure 1 and Table 2). In addition, the highest number of CNV variants at 1684 was on chromosome 2, while chromosome 12 had the lowest at 1026. In terms of CNV length, the average CNV length on chromosome 1 was significantly longer than that on other chromosomes. On chromosome 2, the number of CNVs in the total sample was higher (CNV count > 1600), but the distribution of CNV lengths among individuals was the lowest. These CNVs were distributed across 12 chromosomes, and distributions of deletions and duplications were observed on each chromosome, as shown in Table 3. Most CNVs were duplications, while a small number were deletions. Meanwhile, chromosome 1 contained a large number of duplications, while chromosome 12 had the fewest duplications. Chromosome 1 had the highest count of deletions, while chromosome 2 had the lowest.

**Figure 2:**
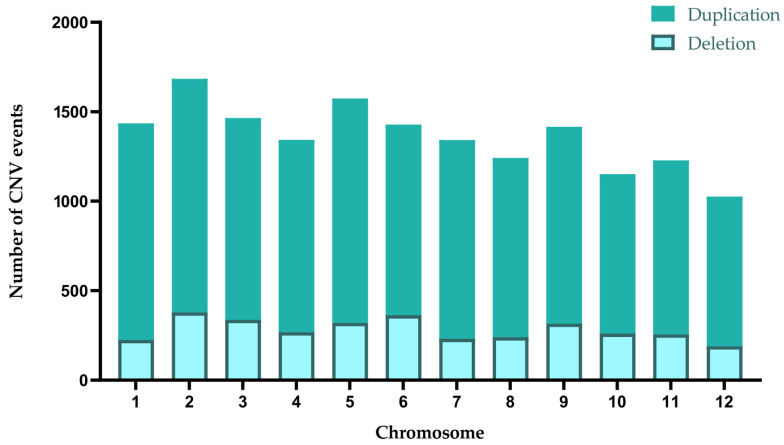
The number of CNV events (deletions and duplications) per chromosome in jujube: each color (bin) represents a different CNV event, and the bars indicate the number of CNVs in each event bin.

For co-observed deletion and duplication events, deletions and duplications showed the highest proportions on chromosomes 1 and 6, respectively. On chromosome 6, 362 deletions were identified among 1428 CNVs, accounting for 35%. Meanwhile, 1210 duplications were found among 1435 CNVs on chromosome 1, representing 84.32% (Table 3 and Figure 2).

### 2.4. Disribution of CNVs on Chromosomes

**Figure 3:**
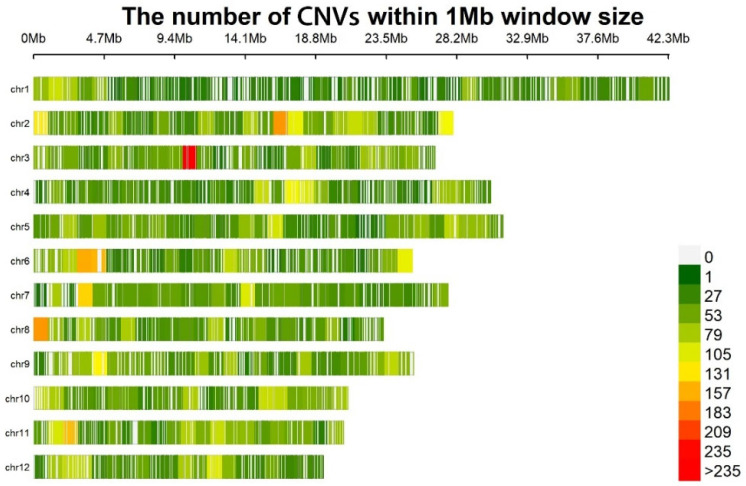
Distribution of CNVs across different chromosomes of the jujube genome within 1 Mb window size. The *x*-axis represents the physical position along each chromosome (from 0 Mb to around 42.3 Mb, varying by chromosome length). The *y*-axis lists the chromosomes. The color scale on the right indicates the number of CNVs within each 1 Mb window: white represents 0 CNVs, and colors from green to red represent increasing numbers of CNVs, with red indicating more than 235 CNVs.

**Figure 4:**
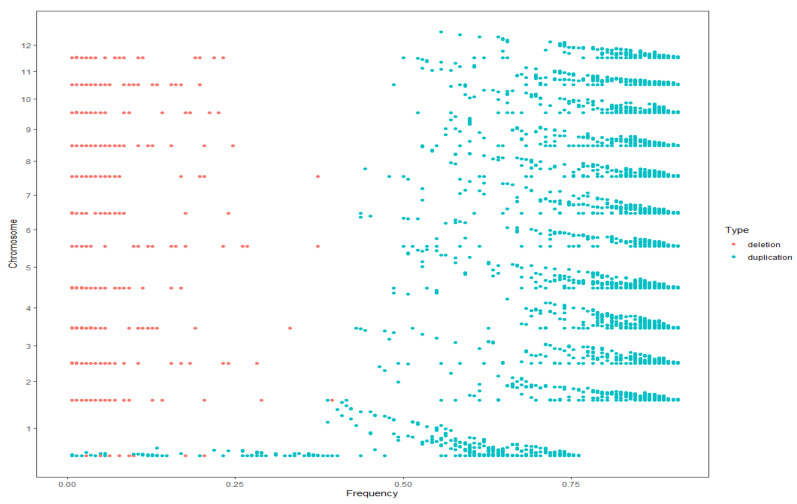
Frequency distribution of CNVs across 12 jujube chromosomes. The plots depict CNVs mapped to the 12 jujube chromosomes (*y*-axis), with the *x*-axis denoting the frequency (expressed as a percentage) of each CNV among 140 accessions. Red and blue points represent deletions and duplications, respectively.

Using the CMplot package in R (R version 4.2.3), we mapped the chromosomal distribution of CNVs in the genomes of the F_1_ population (Figure 3). CNVs were distributed across 12 chromosomes, with numbers varying among chromosomes. The horizontal axis denotes the physical coordinates of chromosomes (in megabase, Mb), while the vertical axis represents chromosomal identifiers (chr1–chr12). Different colors encode the count of CNVs within each 1 Mb sliding window (legend: white = 0 CNVs; red = >235 CNVs). A conspicuously high-abundance cluster (red) was detected within the 9.4–14.1 Mb interval of chromosome 3, where the CNV count per 1 Mb window surpassed 235. Notably, the distribution density of CNVs exhibited no apparent correlation with chromosomal length. For instance, chromosome 1, despite being the longest chromosome, displayed predominantly low CNV counts (green) across most 1 Mb windows, indicative of a sparse distribution pattern. Among the detected CNVs, deletion events existed in the population at a low frequency, and the frequency of duplication events were mostly more than 0.25 (Figure 4).

### 2.5. CNV Analysis of Black Spot Disease Population

**Figure 5:**
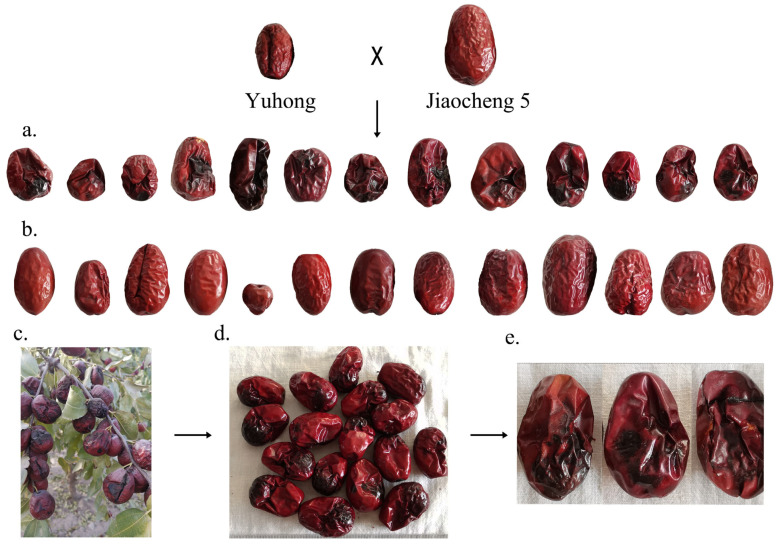
Hybrid offspring of female parent “Yuhong” and male parent “Jiaocheng 5” exhibited severe black spot disease or no black spot disease. (**a**) Thirteen individuals with severe black spot disease; (**b**) thirteen individuals without black spot disease; (**c**) severe black spot disease symptoms on the tree; (**d**) whole-fruit photographs of individuals with severe black spot disease; (**e**) a single individual with severe black spot disease.

Among the 140 individuals of the F1 hybrid population, there were 13 individuals with severe black spot disease and 13 individuals without black spot disease. The phenotype is shown in Figure 5. CNV detection was performed on 13 individuals with severe black spot disease and 13 individuals without black spot disease in the F_1_ hybrid population, respectively. The *t*-test threshold *p* < (0.05/number of all CNV markers) was defined as a significantly differentiated copy number variation region. In order to reduce the false positive rate and avoid possible errors affecting the accuracy of subsequent analysis, we discarded all CNV events less than 1.5 kb [43,44], which was convenient for further analysis of copy number variation related to black spot disease traits.

**Figure 6:**
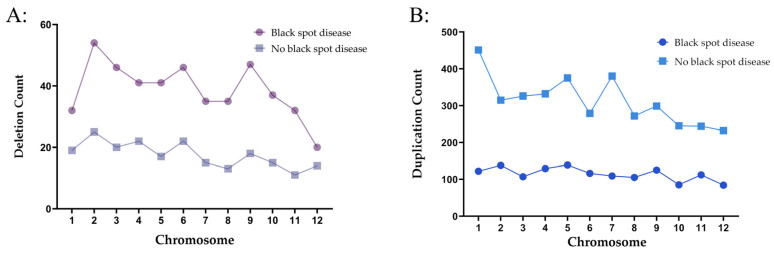
Chromosomal distribution of CNVs in the black spot disease group and the group with no black spot disease. (**A**) Chromosomal distribution of deletion CNVs. (**B**) Chromosomal distribution of duplication CNVs.

Using CNVnator, a software based on the read depth method, we detected a total of 1837 CNVs (including 1371 duplications and 466 deletions) in the 13 individuals with severe black spot disease, with an average length of 2,264,240 bp and a total length of 4,159,409,700 bp. In the group with no black spot disease, 3961 CNVs were detected, including 3750 duplications and 211 deletions, with an average length of 1,030,957 bp and a total length of 4,083,622,500 bp. The chromosomal distribution of CNVs in the severe black spot disease group and the group with no black spot disease is shown in Figure 6. Comparison of chromosome distributions between the severe black spot disease group and the group with no black spot disease showed that the group with no black spot disease had more detected CNVs but their total length and average length were shorter than those in the severe black spot disease group.

### 2.6. Gene Annotation and Enrichment Analysis in CNV Region of Black Spot Disease Population

According to the reference sequence of the jujube genome, this study performed Gene Ontology (GO) functional annotation and KEGG pathway enrichment analysis on CNVs detected on known chromosomes of jujube populations with black spot, which provided value for us to study the relationship between CNV genes and phenotypic variations.

**Figure 7:**
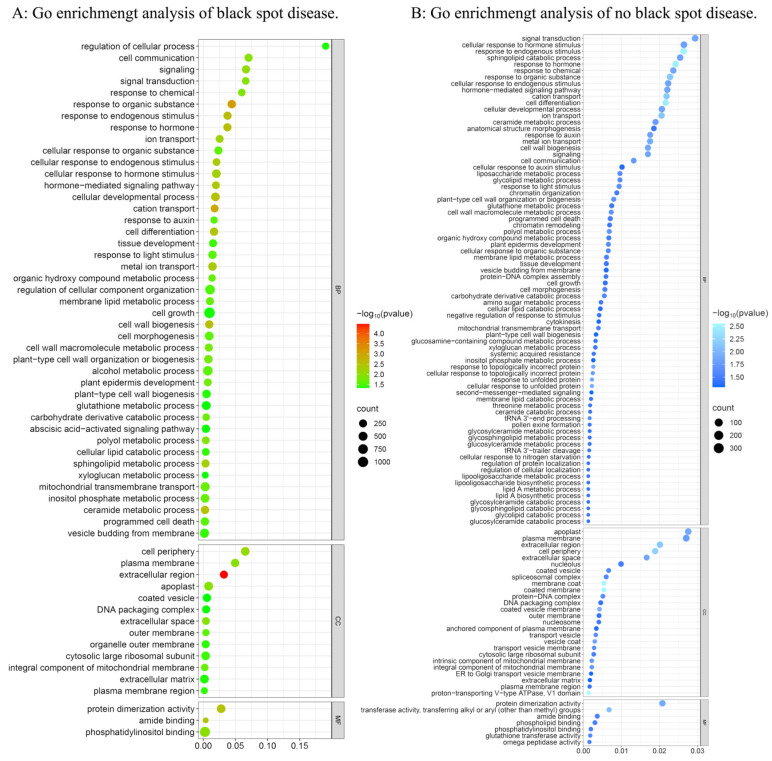
GO enrichment analysis. ((**A**): Go enrichment analysis of black spot disease; (**B**): Go enrichment analysis of no black spot disease).

In addition, the KEGG database was used to determine the pathways showing significant changes (*p* < 0.05). There were five significantly enriched KEGG pathways, which were mainly related to protein processing in the fatty acid degradation (Table 4). The results of GO analysis showed that the expressed genes were divided into three categories: molecular function, cellular composition, and biological process. A total of 59 GO terms were enriched in CNVs with black spot disease (*p* < 0.05). In CNVs without black spot disease, 109 GO terms were enriched. There were 53 terms that were the same. Among the different GO terms, we found a term that was highly related to disease resistance was enriched in CNVs without black spot disease. The GO term is the biological process involved in the systemic acquired resistance (SAR) related pathways, with the GO ID 0009627 (Figure 7).

SAR is an enhanced defense response triggered when plants detect a pathogen. This term contains 23 genes. We annotated these genes and found that eight were associated with disease resistance (Table 5). These genes encoded the negative regulator of systemic acquired resistance *SNI1*, pro-hevein, protein SAR DEFICIENT 4, BTB/POZ domain and ankyrin repeat-containing protein *NPR1*, protein *LIM1*, protein *NIM1-INTERACTING 1* and *NIM1-INTERACTING 2*, and NEGATIVE REGULATOR OF RESISTANCE, participating in the disease resistance process.

**Figure 8:**
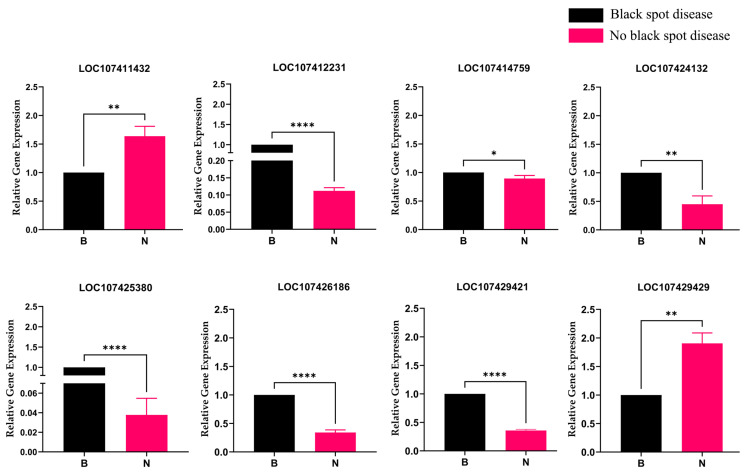
Expression of candidate gene. Black spot disease is represented by black bars, labeled “B”, and no black spot disease is represented by pink bars, labeled “N”. The * above the bars indicates significant differences in gene expression between samples with black spot disease and those without black spot disease (* *p* < 0.05, ** *p <* 0.01, **** *p* < 0.0001).

To investigate the regulatory roles of eight candidate genes in black spot disease in hybrid progenies of jujube, this study used individuals with no black spot disease as experimental materials and individuals with server black spot disease as controls. The expression patterns of these eight genes were analyzed by quantitative real-time PCR (qPCR) (Figure 8). The results showed that the *LOC107411432* (negative regulators *SNI1*) and *LOC107429429* (NEGATIVE REGULATOR OF RESISTANCE (NRR)) were significantly upregulated in healthy plants, whereas the positive regulators *LOC107412231* (pro-hevein), *LOC107414759* (SARD4), *LOC107424132* (NPR1), *LOC107425380* (LIM1), *LOC107426186* (NIM1-INTERACTING 1), and *LOC107429421* (NIM1-INTERACTING 2) were significantly downregulated in healthy plants. This expression pattern is consistent with the core logic of plant disease resistance regulation, reflecting the adaptive regulatory strategy of plants for growth–defense resource allocation in the healthy state.

## 3. Discussion

### 3.1. The Evolution of CNV Detection Methods and the Feasibility of CNV Research in Ziziphus Plants

As an important part of genomic sequence differences, copy number variation has a very important impact on various trait phenotypes [45]. At present, the whole genome-wide detection of CNV detects single nucleotide polymorphism (SNP) chips and arrays comparative genome hybridization (aCGH), and is a broad, economical, and effective method for detecting CNV [10]. However, due to its computational process, it is often affected by low probe density and repetitive sequences, and CNV detection is reduced in the large segment duplication (SD) regions [46]. With the rapid development of whole genome sequencing technology (next generation sequencing, (NGS)), this technology has the advantages of higher coverage and resolution, more accurate copy number estimation, more accurate breakpoint detection, and higher ability to identify new CNVs in the detection of CNV [47]. Read depth (RD) is a detection method in NGS. At present, most of the detections are based on RD. For example, in animal studies such as pig landrace gene copy number variation [48] and bovine genome-wide copy number variation [46], absolute copy number can be detected and it has good performance in detecting large CNVs. CNVnator [42] is a software for detecting CNV based on RD. It detects CNV by statistically analyzing the short read alignment density (RD) of the next-generation sequencing platform. The software can find CNVs of various sizes and their genotyping capabilities throughout the genome, and has strong versatility. It is the most frequently cited software in all CNV detection software [48]. At present, there is a lack of research on CNV in Chinese jujube. In recent years, due to the development of whole genome sequencing technology and the successful construction of the whole genome map of Chinese jujube hybrid offspring [41], it is possible to systematically detect CNV in Chinese jujube at the whole genome level. Based on the next generation sequencing technology and the strategy of genome sequencing read depth (RD) analysis, CNV can be detected in the genome region and the type and number of CNV can be determined. In this study, the whole genome sequencing (WGS) technique was used to construct a genome-wide copy number variation map of Chinese jujube. The whole genome resequencing data of 140 F_1_ individuals of “Yuhong” × “Jiaocheng 5” were used to detect the copy number variation in the whole genome. The average sequencing depth of the parents was 12.43×, while that of the progeny was 2.62×. A total of 203.02 G of raw sequencing data were generated, with an average of 1.43 G per sample. The Q30 base quality ratio reached 92.06%, and the mapping efficiency was 97.09%, indicating high data quality. Although the progeny had low sequencing depth, the high-quality data and large sample size partially compensated for the impact of insufficient depth. Existing studies have shown that, in population genetics research with limited costs, large sample size combined with low sequencing depth is the optimal strategy for achieving highly accurate inferences [49].

### 3.2. Construction of a Genome-Wide CNV Map of Chinese Jujube

In this study, a total of 16,570 CNV events (3607 CNV deletions and 12,963 duplications) were detected in the whole F1 progeny population. There were 118.36 CNV events per individual on average. In view of the fact that it is the first time a genome CNV map of Chinese jujube has been constructed, the results of this study are compared with those of other animal and plant CNV studies. For example, a total of 4715 CNVs were identified in sacred lotus (Nelumbo nucifera), including 448 duplications and 4267 deletions [27]. A total of 253,553 CNVs (244,876 deletions and 8677 duplications) were identified in African goats [17]. Xu et al. [50] identified 914,610 CNVs (838,642 deletions and 75,968 duplications) from 346 apple material. Sakina et al. [51] examined 116 *Malus* accessions (including domesticated apples and their wild relatives) and identified approximately 48,000 segmental duplications and 289 segmental deletions using the RD approach. Cardone et al. [52] detected CNVs in four table grape cultivars using a whole-genome shotgun detection (WSSD) approach modified based on high-throughput sequencing data. The results showed that approximately 9% of the grapevine genome had deletions in at least one cultivar. Furthermore, about 26.13% of the grapevine genome had duplications in at least one cultivar. Furthermore, 46% of the copy number variation regions (CNVRs) overlapped with the segmental duplication regions of the reference genome. The above data and some other research reports show that the range of CNV is very wide. This difference may be due to factors such as the sample size, the CNV detection algorithm, and different sequencing techniques.

### 3.3. CNV Characteristics of Chinese Jujube Individuals Highly Susceptible to Black Spot Disease and Analysis of Related Disease-Resistant Genes

The results showed that 13 individuals with severe black spot disease were selected in the hybrid offspring, and a total of 1837 CNVs (1371 duplicated and 466 deleted) were found in the genome. The average length of CNV was 2,264,240 bp and the total length was 4159.4 Mb. This study identified five pathways significantly associated with black spot disease: fatty acid degradation, glycerophospholipid metabolism, peroxisome, sphingolipid metabolism, and longevity regulating pathway in multiple species. Among them, the two pathways of fatty acid degradation and glycerophospholipid metabolism may be involved in the regulation of black spot disease resistance. During the severe infection stage of *Poacynum hendersonii* by rust pathogens (*Melampsora apocyni*), it was found that most genes in the fatty acid degradation pathway showed a significant upregulation trend, which, together with pathways such as phenylpropanoid biosynthesis and glycerophospholipid metabolism, constituted the core metabolic response of plants to severe disease stress [53]. Gene content annotation and GO functional enrichment analysis of the detected CNVs indicated SAR might be a potential key factor influencing black spot disease resistance. SAR refers to mobile signals emitted by local tissues of a plant when infected by a pathogen, thereby establishing a prolonged broad-spectrum resistance mechanism throughout the plant. Among them, the negative regulator of systemic acquired resistance *SNI1* is a protein in *Arabidopsis thaliana*, which prevents the expression of pathogenesis-related genes (PR) via histone modifications and binding negative cis-acting elements at their promoters [54]. The gene *LOC107411432* encodes the *Ziziphus* jujuba negative regulator of systemic acquired resistance *SNI1*. In Durrant et al.’s study [55], it was found that *SNI1* and *RAD51D* negatively regulated both gene expression and DNA recombination during pathogen infection, thus being involved in short-term defense response and a long-term survival strategy in *Arabidopsis*. *LOC107412231* encodes the *Ziziphus* jujuba pro-hevein. Pro-hevein is a wound-induced protein and a main allergen from the latex of *Hevea brasiliensis* [56]. In 1991, Parijs et al. found that the hevein protein had strong antifungal activity to inhibit several fungi from rubber-tree [57]. In *Arabidopsis thaliana*, both the N-terminal hevein-like domain (CB-HEL) and the C-terminal domain (CD-HEL) in the modular structure of the AtHEL protein exhibited strong antifungal activity, suggesting the inhibition of fungal growth [58]. The hevein proteins were also identified and characterized in barley, and a total of 13 hevein genes were identified. Among them, ChtBD1 domain of barley hevein had the highest antimicrobial activity [59]. *LOC107414759* encodes the *Ziziphus* jujuba protein SAR DEFICIENT 4 (*SARD4*). *SARD4* encodes an enzyme involved in the biosynthesis of pipecolate (Pip), which is a metabolite that coordinates defense amplification and positive regulation of salicylic acid (SA) biosynthesis, and is initiated to ensure effective local resistance induction and SAR establishment [60,61,62]. *LOC107424132* encodes the *Ziziphus* jujuba BTB/POZ domain and ankyrin repeat-containing protein *NPR1*. *LOC107426186* and *LOC10742942* encode the jujube protein *NIM1-INTERACTING 1* and *2*. The NONEXPRESSER OF PR GENES1 (*NPR1*), also called *NIM1* is the central positive regulator of the pathogen defense reaction SAR in *Arabidopsis thaliana*. *NIMIN-1* and *NIMIN-2* encode structurally related proteins interacting physically with *NPR1/NIM1*. When the plants are challenged by pathogens in *Arabidopsis*, the *NIMIN* proteins play a important role to physically interact and modify activity with *NPR1/NIM1* [63]. *LOC107425380* encodes the jujube protein *LIM1*. In the early study, the LIM protein, *GhWLM1C* in upland cotton (*Gossypium hirsutum*) responded to the infection of the cotton fungal pathogen *Verticillium dahliae* (*V. dahliae*), and resistance was reduced by silencing the *GhWLM1C* [64]. This demonstrates the function of *LIM1* protein in plant resistance. *LOC107429429* encodes the jujube protein NEGATIVE REGULATOR OF RESISTANCE (NRR). NRR protein interacts with NPR1 in the NPR1-interacting domain (NI25) and acts as a negative regulator of disease resistance.

In this study, eight genes annotated as systemic acquired resistance (SAR)-related were identified in hybrid progenies of *Ziziphus jujuba*. As key components of the plant disease-resistant molecular network, these genes cover positive and negative regulators, core signal transduction proteins, and effector molecules of defense responses in the SAR pathway, and may play important roles by synergistically regulating the multi-level immune responses of plants against pathogen invasion. Among them, the negative regulators *LOC107411432* (SNI1) and *LOC107429429* (NRR) may maintain the growth–defense balance in healthy plants by inhibiting the overactivation of the SAR pathway. In contrast, positive regulators such as *LOC107412231* (pro-hevein) and *LOC107414759* (SARD4) may respond rapidly to pathogen invasion, enhancing plant defense capabilities by activating the synthesis of antimicrobial molecules and promoting the transmission of systemic resistance signals. The core regulator *LOC107424132* (NPR1) and its interacting proteins *LOC107426186* (NIM1-INTERACTING 1) and *LOC107429421* (NIM1-INTERACTING 2) may act as signal hubs to integrate defense signals such as salicylic acid and regulate the precise expression of downstream pathogenesis-related genes, while *LOC107425380* (LIM1) may participate in the cascade amplification of defense signals by mediating protein interactions. We speculate that these eight genes may function by deeply participating in the regulation of the SAR signaling pathway. Future studies need to further clarify their specific mechanisms of action in jujube black spot resistance through functional verification experiments such as gene knockout and overexpression, combined with techniques like protein interaction analysis and transcriptome analysis. This will provide available genetic resources and a theoretical basis for the molecular breeding of jujube black spot resistance, promoting the precise breeding of disease-resistant varieties.

## 4. Materials and Methods

### 4.1. Plant Materials and Growth Conditions

The female parent is named “Yuhong”, which is a male-sterile cultivar Chinese jujube with the advantages of good quality, high yield, and strong adaptability. The male parent is called “Jiaocheng 5”, which is a superior line of “Junzao” and has strong disease resistance. We crossed “Yuhong” and “Jiaocheng 5”, and produced 140 Chinese jujube hybrids for the experiment. All experimental materials were planted in Alar City, Xinjiang, China (81°28′ E, 40°59′ N). Alar City has a warm temperate extreme continental arid desert climate, with long sunshine hours, which is suitable for jujube cultivation. The plant spacing was set at 0.4 m × 0.6 m, with a density of 10–15 plants per row, and conventional water and fertilizer management was applied to the experimental site. During the growing season, fruit trees were pruned from May to July. Insecticide for red spider was applied 2 times a year to manage diseases and fruit damage. We collected leaves from 140 jujube cross progeny and parents and froze them in a −80 °C freezer for subsequent experiments. The experiment was replicated three times, and the final results were averaged.

### 4.2. DNA Extraction

Genomic DNA was extracted from the healthy young leaves (second or third leaf from the apex of the bearing shoot, less than 1 cm^2^) of parents and each individual progeny plant (F1 generation) using a modified CTAB method [65]. The purity of the DNA was measured using a NanoDrop 2000 microspectrophotometer (Thermo Fisher Scientific, Beijing, China), and the DNA concentration was measured using PicoGreen of each sample. Finally, DNA purity and band integrity were analyzed using agarose gel electrophoresis at a concentration of 1%.

### 4.3. Whole Genome Resequencing

After passing DNA quality control, ultrasonic waves were used to randomly fragment the DNA. The fragmented DNA was sequentially end-repaired and A tails and DNA fragment connectors were added, after which the fragments with a genome length of about 400 bp were enriched by magnetic beads, and the sequencing libraries were formed by PCR amplification.

Then, when the quality inspection of the library was qualified, the Illumina HiSeq^TM^ platform was used for genome sequencing (The sequencing was performed by Majorbio Bio-pharm Technology Co., Ltd., Shanghai, China). The sequencing strategy was Illumina PE150, which was sequenced at 300 bp. The sequenced DNA sequence was uploaded to the NCBI database (registration number is PRJNA877294) and was also used in our previous publication on the genetic mapping of the whole genome of Chinese jujube [41].

### 4.4. Sequencing Data Processing and Read Alignments

The Illumina Hiseq^TM^ sequencing of the data (raw data) was offline, and quality control was performed on the offloaded data to filter out the low data and obtain high quality data (clean data). The clean data were then compared with the jujube reference genome “https://www.ncbi.nlm.nih.gov/genome/?term=Ziziphus+jujuba, accessed on 6 February 2015” using BWA software (version: 0.7.17-r1188) [66] to obtain position attribution of the sequence. The BAM files were corrected using GATK’s Best Practices process [67].

### 4.5. Mapping of CNVs

CNVnator [42] software was used to detect the structural variation of CNVs for subsequent analysis in the jujube genome samples. The bin size for RD analysis in the CNVnator software was set to 500 bp. CNVnator can accurately identify the CNV information of each individual. CNVs with *p*-values calculated using *t*-test statistics less than 1 were screened for mapping construction. The CMplot package of R software (R version 4.2.3) was used to construct a genetic map of CNVs of jujube cross progeny and to visualize and analyze the genetic map. The serial number of each chromosome was used as the vertical coordinate, and the length of each chromosome was used as the horizontal coordinate.

### 4.6. Definition and Association Analysis of Regions of Variation in Copy Number of Jujube Black Spot

In the identification process of CNVnator, the significance of the difference between the sequencing depth of CNV region and the average sequencing depth of the whole genome was expressed as *p* (multivariate hypothesis *t* test). We discarded all CNVs with *p* > 0.05, and only retained high-confidence CNV recognition results. Considering that the lower sequencing depth may reduce the ability and reliability of detecting small fragment CNV based on the RD method [42,43], in order to reduce the false positive rate and avoid possible errors affecting the accuracy of subsequent analysis, we discarded all CNV events less than 1.5 kb and only used the longer fragments for further analysis [44]. At the same time, 13 individuals with severe black spot disease were compared with 13 individuals without black spot disease. GraphPad Prism 8 software was used to analyze the association of the detected CNV with black spot trait, and to find the CNV segments that significantly affected the black spot trait.

### 4.7. Gene Annotation and Functional Enrichment Analysis of CNV in Black Spot Disease Groups

Genes covered by the CNV region were found and Gene Ontology (GO) and Kyoto Encyclopedia of Genes and Genomes (KEGG) functional annotation was performed using Gene Ontology Resource and Kyoto Encyclopedia of Genes and Genomes databases. GO and KEGG enrichment analysis of the genes involved in the region was performed using the R package clusterProfiler (R version 4.2.3) [68]. Based on the non-redundant (NR) protein sequence database, the functional information was annotated; and meanwhile the function of the relevant gene was also searched in conjunction with the National Center for Biotechnology Information (NCBI) website gene database to find the literature on the reported function of the gene.

### 4.8. RT-qPCR Analysis

In this study, total RNA was extracted from fruits using the TIANGEN RNAprep Pure Plant Plus Kit (polysaccharide- and polyphenolics-rich) (TIANGEN BIOTECH (BEIJING) Co., Ltd., Beijing, China). cDNA was synthesized via reverse transcription using the Vazyme Reverse Transcription Kit HiScript^®^ II Q RT SuperMix for qPCR (+gDNA wiper). RT-qPCR assays were performed on a real-time PCR instrument (BIOER QuantGene 9600 Real-Time PCR Detection System) (Hangzhou Bioer Technology Co., Ltd., Hangzhou, China). The RT-qPCR reaction mixture consisted of 5 μL SYBR green dye, 3.6 μL RNase-free water, 1 μL cDNA, 0.2 μL forward primer, and 0.2 μL reverse primer, with a total reaction volume of 10 μL. The RT-qPCR amplification program was as follows: pre-denaturation at 95 °C for 4 min, followed by 40 cycles of denaturation at 95 °C for 15 s, and annealing and extension at 58 °C for 30 s. The relative expression levels of genes were calculated using the 2^−∆∆Ct^ method. The gene expression level of diseased varieties was set as the reference (designated as 1), and the relative expression levels of non-diseased varieties were calculated relative to those of diseased varieties. The *Zjactin* gene was used as the internal control for RT-qPCR analysis.

## 5. Conclusions

This study is the first to conduct a genome-wide copy number variation (CNV) analysis of Chinese jujube (*Ziziphus jujuba*) and construct a CNV-based genetic map of jujube. In this study, we identified 16,570 CNVs from “Yuhong” × “Jiaocheng 5” and 140 hybrid progeny materials, among which 3607 CNVs were deletion type and 12,963 CNVs were duplication type. Thirteen individuals with severe black spot disease were selected in the hybrid offspring, and a total of 1837 CNVs (1371 duplicated and 466 deleted) were found in the genome. By comparing CNVs between jujube plants with black spot disease and normal jujube plants, we identified eight candidate genes that may affect jujube black spot disease. After validation by qPCR, these results further support their potential role in regulating black spot disease resistance. This study provides valuable insights into understanding the genetic basis of phenotypic variation in jujube and lays a foundation for jujube genetic breeding.

## Figures and Tables

**Table 1:** CNV distribution types.

Cultivars	Number of CNVs	Deletion	Duplication	Range of Length/bp
Yuhong	2646	1134	1512	1–42,213,900
Jiaocheng 5	2533	1045	1488	1–42,390,600
F_1_	16,570	3607	12,963	1–42,390,600
Average of F_1_	118.36	25.76	92.59	1–42,390,600

**Table 2:** Distribution of CNV on chromosomes in jujube.

Chromosome	Length of Chromosome/bp	Number of CNVs	Average Length/bp
1	42,390,600	1435	29,540.49
2	27,987,000	1684	16,619.36
3	26,737,500	1465	18,250.85
4	30,445,800	1343	22,669.99
5	31,365,600	1574	19,927.32
6	25,260,000	1428	17,689.08
7	27,644,400	1342	20,599.40
8	23,351,100	1242	18,801.21
9	25,348,800	1416	17,901.69
10	20,983,500	1151	18,230.67
11	20,704,200	1228	16,860.10
12	19,346,100	1026	18,855.85

**Table 3:** The proportion of jujube CNV on chromosomes.

Chromosome	Deletion	Duplication	Deletion/ Duplication	Deletion/CNVs	Duplication/CNVs
1	225	1210	0.19	15.68%	84.32%
2	378	1306	0.29	22.45%	77.55%
3	337	1128	0.30	23.00%	77.00%
4	267	1076	0.25	19.88%	80.12%
5	319	1255	0.25	20.27%	79.73%
6	362	1066	0.34	25.35%	74.65%
7	231	1111	0.21	17.21%	82.79%
8	240	1002	0.24	19.32%	80.68%
9	315	1101	0.29	22.25%	77.75%
10	260	891	0.29	22.59%	77.41%
11	255	973	0.26	20.77%	79.23%
12	189	837	0.23	18.42%	81.58%

**Table 4:** Enriched KEGG pathway.

ID	Term
ko00071	Fatty acid degradation
ko00564	Glycerophospholipid metabolism
ko04146	Peroxisome
ko00600	Sphingolipid metabolism
ko04213	Longevity regulating pathway—multiple species

**Table 5:** Candidate gene functional predictions.

Gene ID	Function Annotation
*LOC107411432*	negative regulator of systemic acquired resistance SNI1
*LOC107412231*	pro-hevein
*LOC107414759*	protein SAR DEFICIENT 4
*LOC107424132*	BTB/POZ domain and ankyrin repeat-containing protein NPR1
*LOC107425380*	protein LIM1
*LOC107426186*	protein NIM1-INTERACTING 1
*LOC107429421*	protein NIM1-INTERACTING 2
*LOC107429429*	protein NEGATIVE REGULATOR OF RESISTANCE

## Data Availability

All relevant data can be found within this paper. And the sequenced DNA sequence was uploaded to the NCBI database (registration number is PRJNA877294) and was also used in our previous publication on the genetic mapping of the whole genome of jujube [47].
